# "Opticians" and "Sight-Testing"

**Published:** 1903-05-23

**Authors:** 


### The Hospital.

A Journal of

The Medical Sciences and Hospital Administration.

Edited by Sir Henry Burdett, K.C.B.

Vol. XXXIV.—No. 869. [May 23, 1903.

### "Opticians" and "Sight-testing."

A very strenuous endeavour has been made, now for some years past, to establish in the estimation of the public a new craft, that of so-called "opticians" or spectacle makers, who profess to understand the derangements of vision, and to be able to prescribe for them in a correct and satisfactory manner. One of the City companies has, we believe, actually instituted examinations for people of this class; and we have lately seen a circular, issued by the "British Optical Association," whatever that may be, in which further steps in the same direction are asked for and recommended. The Association desires also to promote the cultivation of "optology," a delicious coinage which reminds us of another of a somewhat similar character. Many years ago, a suburban physician published a book called "Glossology," in which he endeavoured to connect certain localities on the surface of the tongue with certain internal organs, and thus to reduce diagnosis to an art of extreme simplicity. The tongue was mapped out, something after the fashion of the "phrenological" mapping out of the cranium, here a place for the heart, here for the pancreas, here and there for the left and the right kidneys respectively. A domestic servant in the vicinity, who consulted the medical attendant of the family in which she lived, casually mentioned to him that her mother was not well. "Oh," said the doctor, "you had better tell her to come round and see me." "No, thank you, sir," was the reply, "mother always goes to Dr. R. She is a *glopologist.*" One of the "optological" circulars which we have seen dwells upon the case of a qualified medical practitioner, who is alleged to have been unable to see the optic disc with the ophthalmoscope, to have pronounced a case of cataract to be iritis (or *vice versâ*, we forget which), to have declared vision of $\frac{6}{18}$ths to be "normal," and to have committed other atrocities, against which, the inference is, a qualified "optologist" would have been secure. In other words, a gang of incredibly impudent pretenders are endeavouring to persuade the public that persons possessing no medical knowledge may be safely trusted to prescribe for the derangements of one of the most sensitive and most important organs of the body; and they are beginning to adorn their shop windows with legends which unquestionably sail very close to the wind in the adoption of titles calculated to lead to the impression that they are qualified medical practitioners. Their names are followed by fragmentary alphabets of large proportions, and they exhibit framed "diplomas" bearing a very close general resemblance to those which were formerly given to midwives by the Obstetrical Society, and which were altered or abandoned under pressure from the General Medical Council. It seems to be high time for that body to take into consideration the words and acts of the "optologists." It has, of course, no direct authority over them, but it might at least take steps which would prevent their pretensions from being conceded through default of opposition.

The claim of spectacle makers to prescribe for defects of vision is, of course, as absurd as would be a claim on the part of microscope makers to be experts in bacteriology, or a claim on the part of crutch makers to be "specialists" in lameness. It is not only futile, on the ground recently taken by a contemporary, that no spectacle maker has ever contributed in any way to our knowledge of the nature and causes of visual defects, but it is at once put out of court by the consideration that the spectacle maker, whatever the nature of the malady, has no resource but spectacles, just as the crutch maker would have no resource but crutches. He might rearrange the handles to give a more convenient grip, or he might alter the padding under the arms, but he would not know when amputation was expedient, and he would not be able to perform it. It is quite possible, of course, that there may be registered medical men in practice who have not paid as much attention to eye work as they should have done, and who are less qualified to advise concerning vision than they should be; but this does not advance the case of the "optologists" one whit, or give even the slightest increase of colour to their pretensions. The essential point is that no one who does not possess medical knowledge can ever, in any circumstances, be qualified to advise concerning conditions which affect vital organs, and are liable to be either caused or complicated by disease; and to this general and universally true statement the eyes offer no exception, even although their functions are dependent upon purely physical as well as upon vital endowments. It applies to them, in fact, even more than to other parts of the body, because the diseases of the eye, as a living organ, are liable to be obscured, to the apprehensions of a non-medical observer, by its purely optical derangements. If spectacle sellers, as a rule, continue in the course upon which some of them are now entering; if they, to quote the words of Donders, "rendered rash by ignorance," begin to use the ophthalmoscope, to examine the interior of the eye, to test refraction by retinoscopy, to measure the relative strengths of the ocular muscles and to prescribe prisms for the correction of supposed abnormalities, they will assuredly, before long, land their customers in disaster which must ultimately recoil upon their own heads. As soon as they step beyond the simplest cases of presbyopia, they will enter upon ground beset by snares and pitfalls, and the consequences likely to follow from their inevitable ignorance will only be equalled by the dishonesty of their pretensions to knowledge which it is impossible for them to possess.

